# Insight On Colorectal Carcinoma Infiltration by Studying Perilesional Extracellular Matrix

**DOI:** 10.1038/srep22522

**Published:** 2016-03-04

**Authors:** Manuela Nebuloni, Luca Albarello, Annapaola Andolfo, Cinzia Magagnotti, Luca Genovese, Irene Locatelli, Giovanni Tonon, Erika Longhi, Pietro Zerbi, Raffaele Allevi, Alessandro Podestà, Luca Puricelli, Paolo Milani, Armando Soldarini, Andrea Salonia, Massimo Alfano

**Affiliations:** 1 L. Sacco Hospital, Department of Biomedical and Clinical Sciences, University of Milan, 20157 Milan, Italy; 2 Department of Pathology, San Raffaele Scientific Institute, Milan, Italy; 3 ProMiFa, Protein Microsequencing Facility, San Raffaele Scientific Institute, Milan, Italy; 4 Division of Experimental Oncology/Unit of Urology, URI, IRCCS Ospedale San Raffaele, 20132 Milan, Italy; 5 Functional Genomics of Cancer Unit, Division of Experimental Oncology, Istituto di Ricovero e Cura a Carattere Scientifico (IRCCS) San Raffaele Scientific Institute, Milan, Italy; 6 L. Sacco Hospital, Department of Biomedical and Clinical Sciences, Centro di Microscopia Elettronica per lo studio delle Nanotecnologie Applicate alla medicina “C.M.E.N.A.”, University of Milan, 20157 Milan, Italy; 7 Interdisciplinary Centre for Nanostructured Materials and Interfaces (CIMaINa) and Dept. of Physics, Università degli Studi di Milano, Milano, Italy; 8 Diagnostica e Ricerca San Raffaele, Milan, Italy; 9 Università Vita-Salute San Raffaele, 20132 Milan, Italy; 10 Research Doctorate Program in Urology, Magna Graecia University, 88100 Catanzaro, Italy

## Abstract

The extracellular matrix (ECM) from perilesional and colorectal carcinoma (CRC), but not healthy colon, sustains proliferation and invasion of tumor cells. We investigated the biochemical and physical diversity of ECM in pair-wised comparisons of healthy, perilesional and CRC specimens. Progressive linearization and degree of organization of fibrils was observed from healthy to perilesional and CRC ECM, and was associated with a steady increase of stiffness and collagen crosslinking. In the perilesional ECM these modifications coincided with increased vascularization, whereas in the neoplastic ECM they were associated with altered modulation of matrisome proteins, increased content of hydroxylated lysine and lysyl oxidase. This study identifies the increased stiffness and crosslinking of the perilesional ECM predisposing an environment suitable for CRC invasion as a phenomenon associated with vascularization. The increased stiffness of colon areas may represent a new predictive marker of desmoplastic region predisposing to invasion, thus offering new potential application for monitoring adenoma with invasive potential.

Tumor pathogenesis is affected by genetic mutations, escape from recognition by the immune system and modifications of the extracellular environment. Among the latter, transformed cells require the existence, generation and recruitment of a microenvironment permissive for tumor growth, the spread of neoplastic cells into the blood vasculature and/or lymphatic system, and seeding in distant organs^1^. Each of these tumorigenic steps is fine-tuned by factors related to the tumor cells and host^2^. As a major component of the local microenvironment, the extracellular matrix (ECM) has emerged as an active participant in major cell behaviors, including developmental processes and various stages of the carcinogenic process. Indeed, certain stroma components (i.e. vasculature) play a tumor promoting role^3^ while others (i.e., myofibroblasts) have a tumor-suppressive role^3^,^4^.

Dysregulation of the biochemical and physical features of the extracellular matrix such as composition, architecture, ultrastructural 3D conformation or stiffness of the ECM are associated with a lack of asymmetric division and differentiation of stem cells, epithelial-mesenchymal transition of cancer cells, as well as the modulation of cell migration, differentiation and proliferation sustaining the onset and progression of cancer both at primary and metastatic sites^5^,^6^,^7^,^8^. As for other epithelial cancers resulting from aberrant epithelial-mesenchymal interactions^8^ the ECM profoundly regulates CRC progression and metastasis. Colon adenoma-carcinoma progression is associated with an overexpression of collagen XII^9^, whereas liver metastasis is preceded by an accumulation of collagen IV in the liver where the conditioned hepatic ECM has to mediate mesenchymal-epithelial transition^10^,^11^. Not just biochemical composition but also increased lysyl oxidase (LOX) dependent crosslinking and stiffness have recently been reported to be responsible for fibrosis enhanced metastatic colonization of breast and colon cancer cells^10^,^11^,^12^,^13^,^14^.

An important area of future cancer research will be to determine whether an abnormal ECM could be an effective cancer therapeutic target. To achieve this goal, we must understand how the ECM composition and organization are normally maintained and regulated and how they may be deregulated in cancer. A daunting task in this regard will be to determine which ECM changes have causative effects on disease progression and how these changes, alone or in combination with others, may affect cancer cells and cells in the stromal compartment^6^. In particular, discriminating which among the many features of the ECM is mandatory for invasion of the surrounding matrix is of priority.

We recently reported that the ECM from healthy colon mucosa constrains the spreading of metastatic cells, thus indicating the contribution of host factors versus the intrinsic capacity of neoplastic cells to invade the matrix. On the contrary the ECM from perilesional mucosa and CRC supported cell infiltration and increased cell proliferation^15^. The aim of this study was to unveil the features of the ECM in the perilesional mucosa mandatory for tumor infiltration. We investigated biochemical and mechanical features of the ECM isolated from pair-wised healthy colon mucosa, perilesional mucosa and infiltrated CRC, and identified a steady increase of crosslinking and stiffness from healthy to perilesional to CRC ECM. This study also identifies two different mechanisms associated with the increased stiffness occurring in the perilesional and CRC ECM, such as increased vascularization in the perilesional area and increased content of hydroxylysine in the CRC ECM.

## Methods

### Patients and tissue specimens

Patients that underwent colon surgical resection at Ospedale San Raffaele (Milan, Italy) were included in this study. Matched specimens were collected from the left colon of patients undergoing resection surgery for sporadic CRC and obtained through the Unit of Surgical Pathology (Ospedale San Raffaele, Milan, Italy). All patients that participated in this study provided written informed consent. All experimental protocols were approved by the Institutional Review Board (Authorization protocol 279/DG, Ethic Committee Ospedale San Raffaele, Milan, Italy), and all methods were carried out in accordance with the approved guidelines. All mucosa specimens encompassed the luminal surface, mucosa and upper submucosa. Neoplastic, peritumoral and healthy areas were collected from fresh and unfixed surgical specimen within one hour after surgery.

Neoplastic tissue was obtained at the edge of infiltrating neoplasia, and healthy colon mucosa was from the resection margins (>10 cm far from the CRC).

We classified perilesional tissue based on lack of epithelial dysplasia, mild architectural abnormalities and blood vessels with elongated and dilated shape. All the perilesional areas used in the study were in a range of 0.5–1 cm far from the edge of the neoplastic lesion.

Each specimen was divided in two parts for evaluation of histology and preparation of ECM.

Histological report with tumor histotype, staging and grading was performed by pathologists on the original surgical specimen (Supplementary Material 1) according to TNM Classification of Malignant Tumours, 7^th^ Edition (2009).

### ECM purification and protein identification using nanoLiquid-Chromatography MS/MS

Tissues were collected from paired healthy colon, perilesional area and CRC, and tissue-derived ECM prepared as previously described^15^. ECMs were weighed, cut in small pieces and dissolved (1/5 weight/volume) in a buffer containing 5 M urea, 2M thiourea, 2% CHAPS, 2% Zwittergent and 10 μl/ml protease inhibitors using a plastic potter. After 24 h shaking at 1400 rpm at room temperature, the samples were centrifuged at 14000 rpm at 4 °C for 15 minutes. The recovered supernatant was analyzed to determine total protein concentration using BioRad protein assay and BSA as standard. Forty μg of total protein from each sample were in-solution digested using Filter Aided Sample Preparation (FASP) protocol as reported in literature^16^. Samples were desalted using Stage tips C18 columns (ThermoScientific) and injected in a capillary chromatographic system (EasyLC, Proxeon Biosystem). Peptide separations occurred on a home-made 25 cm reverse phase spraying fused silica capillary column, packed with 3-μm ReproSil 120 Å C18 AQ. A gradient of eluents A (pure water with 2% v/v ACN, 0.5% v/v acetic acid) and B (ACN with 20% v/v pure water with 0.5% v/v acetic acid) was used to achieve separation (0.15 μL/min flow rate) (from 10 to 35% B in 230 minutes, from 35 to 50% B in 5 minutes and from 50 to 70% B in 30 minutes). MS analysis was performed using an LTQ-Orbitrap mass spectrometer (ThermoScientific) equipped with a nanoelectrospray ion source (Proxeon Biosystems). Full scan mass spectra were acquired with the lock-mass option and resolution set to 60,000. The acquisition mass range for each sample was from *m*/*z* 300 to 1750 Da. The ten most intense doubly and triply charged ions were selected and fragmented in the ion trap using normalized collision energy 37%. Target ions already selected for the MS/MS were dynamically excluded for 120 seconds. All MS/MS samples were analyzed using Mascot (v.2.2.07, Matrix Science, London, UK) search engine to search the UniProt_Human Complete Proteome_ cp_hum_2015_01. Searches were performed with trypsin specificity, two missed cleavages allowed, cysteine carbamidomethylation as fixed modification, acetylation at protein N-terminus, oxidation of methionine and lysine as variable modifications. Mass tolerance was set to 5 ppm and 0.6 Da for precursor and fragment ions, respectively. To quantify proteins, the raw data were loaded into the MaxQuant^17^ software version 1.3.0.5: label-free protein quantification was based on the intensities of precursors, both as protein intensities and normalized protein intensities (LFQ intensities). Peptides and proteins were accepted with a FDR less than 1%, two minimum peptides per protein with one unique. The experiments were performed in technical triplicates, with technical reproducibility among replicates (both as number of unique peptides and LFQ intensities for each protein) >0.98. The proteins identified by proteomic analysis were compared to the “*Total Human Matrisome”* database (http://web.mit.edu/hyneslab/matrisome/)^18^ in October 2013, when the database comprised 1065 genes coding for human proteins in the extra-cellular matrix.

### Histological and immunohistochemistry analysis

Cells and nuclei in the tissues and ECMs were evaluated by hematoxylin-eosin, and cellular antigens (Tenascin and ER-b) by immunohistochemistry, as reported^15^,^19^. Antibody against ER-b receptor was the clone EME02 (Novocastra, Leica Biosystems Newcastle, UK), as reported^20^. Goat anti-human Matrilin-2 Ab was the clone 3044-MN from, R&D Systems (MN, USA). Mouse monoclonal anti-tenascin Ab (clone ab58954) and rabbit monoclonal anti-LOX (clone EPR4025) were from Abcam; anti-CD34 mAb (clone QBEnd/10 from Ventana Medical Systems, Arizona, USA) was used for blood vessels. Number and width of capillaries were measured by using the plug-in “cell counter” and the function “measure” in the Image J software (version 1.5)^21^ on high magnification images.

### Western blot

Tissues and ECMs were weighed, cut in small pieces and dissolved (1/5 weight/volume) in a buffer containing 5 M urea, 2 M thiourea, 2% CHAPS, 2% Zwittergent and 10 μl/ml protease inhibitors using a plastic potter. After 24 h shaking at 1400 rpm at room temperature, the samples were centrifuged at 14000 rpm at 4 °C for 15 minutes. From recovered supernatant the buffer was exchanged against PBS by using the Amicon Ultra-0.5 ml device (Merck Millipore, Darmstadt, Germany), and total protein concentration estimated using BioRad protein assay and BSA as standard.

Level of Matrilin-2 expression was evaluated in pair-wised ECMs and tissues; 20 μg of ECM and 50 μg of tissue lysates were loaded onto 8% SDS-PAGE and Matrilin-2 revealed by goat-anti human Matrilin-2 (clone 3044-MN, R&D Systems, MN, USA).

Level of LOX expression was evaluated in pair-wised ECMs. Fifteen μg of lysate were loaded onto 12% SDS-PAGE and LOX revealed by mouse monoclonal anti lysyl oxidase Ab (clone 8G5, Lifespan Biosciences, Seattle WA, USA). Mouse monoclonal anti-collagen III antibody (clone FH-7A) was from Abcam.

### Nanoindentation measurements by atomic force microscopy (AFM)

The AFM analysis was carried out on ECMs derived from healthy colon, perilesional area and CRC of three patients. ECMs were grossly dried and attached to glass coverslips (diameter 15 mm) by means of a thin bi-adhesive tape. ECMs were then attached to the bottom of Petri dishes (Greiner Bio-One) and left overnight in an evacuated desiccator in order to dry out and improve spreading and adhesion on the substrate. Prior to AFM measurements, the Petri dish hosting the ECM sample was filled with PBS buffer and the ECM was allowed to rehydrate for 30 minutes at room temperature. Measurements were carried out at room temperature. Hydration-dehydration processes could in principle modify the pristine rigidity of ECMs; with this in mind, we have strictly followed the same protocol for the preparation of each ECM sample, in order to be sure that the results obtained on different samples could be compared.

For the measurement of the Young’s modulus of ECM samples, a Bioscope Catalyst AFM (Bruker) was used to collect series of force vs distance curves^22^,^23^. We have used monolithic borosilicate glass probes consisting in micrometer-sized spherical glass beads with radius R = 8–10 μm attached to silicon cantilevers with force constant k = 0.2–0.3 N/m. Probes were produced according to an established custom protocol^24^. The probes’ diameter was chosen in order to have a reliable and robust mechanical readout, capturing the overall elastic behavior of ECM on a scale comparable to the size of its characteristic micro-structural domains, as observed in scanning electron microscopy images reported in this manuscript.

Each set of force curves (a force volume) consisted of a 16 × 16 array of curves acquired on a 70μm × 70*μ*m area. Ten force volumes were typically recorded on each ECM sample, on macroscopically separated regions, with the exception of patient #1, where only a few hundreds force curves were acquired as a preliminary test.

All the measurements were performed with the following parameters: 4096 points per curve, ramp length L = 10 μm, maximum applied force F = 60–70 nN, and ramp frequency f = 1.1 Hz. Typically, indentations up to 2–3 μm were obtained. Data processing of force volumes was carried out in Matlab environment according to the published protocol^23^. The values of the Young’s modulus were extracted by fitting the Hertz model to each indentation curve. A first very soft indentation region (0–35% of total indentation) was excluded, in order to separate the possible contribution of loosely-bound superficial layers.

The cumulative distributions of Young’s modulus values of the ECMs from the three donors turned out to be the envelop of several nearly lognormal modes, representing the major contributions to the overall ECM rigidity and originating from micro-scale domains that the AFM probe was able to resolve. Multi-Gaussian fit in semilog10 scale allowed identifying the peak value E’ and the geometric standard deviation 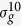 of each lognormal mode; from these values the median value E_med_ and the standard deviation of the median σ_med_ were evaluated for all modes^25^ as


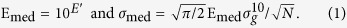


N being the number of force curves in each mode (typically N = 1000–2000). The effective rigidity of each ECM sample was characterized by the weighted average of median values


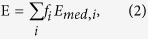


using the fraction f = N/N_tot_ of force curves in the mode as weight; the total error σ_E_ associated to E was calculated by summing in quadrature the propagated error of the medians


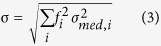


and an effective instrumental relative error


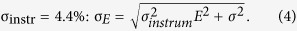


The average median values of the Young’s modulus of the healthy, perilesional and CRC ECM of all donors have also been evaluated; the corresponding error has been calculated as the standard deviation of the mean.

### Collagen and crosslinking

ECM was hydrolysed in 1 ml of 6 M HCl for 20 h at 110 °C, and the acid lysate added of internal standard control and assayed for hydroxyproline content by HPLC (Hydroxyproline reagent kit #195–9501, Biorad); the amount of collagen was estimated based on the level of hydroxyproline accounting for 13.5% of the collagen amino acid composition^26^. Hydroxylysylpyridinoline (HP) and Lysylpyridinoline (LP) were measured by HPLC, in ECM hydrolised in 0.5 ml of 6 M HCl for 20 h at 110 °C, and added of internal standard (Crosslinks, pyridinolin and deoxypyridinolin kit, Chromsystem kit #48000).

### Scanning electron microscopy

Pair-wised tissues were collected from CRC patients, selecting areas of mucosa, submucosa, muscularis propria and subserosa from healthy colon and neoplasia. Pieces of tissue were obtained by performing punch biopsy using a sterile cork borer into tissue, producing a cylindrical core of tissue (2 mm diameter and 5 mm length). ECMs were prepared as previously described^15^. Briefly, ECM were fixed with 2.5% glutaraldehyde (25% solution, electron microscopy grade) in PBS for 24 hours, then dewaxed, dehydrated in an ascending degree of ethanol (10–25–50–75–90–100%) and dried overnight in hexamethyldisilizane. ECMs were coated with gold-palladium after evaporation of hexamethyldisilizane and examined in a Leica S420 scanning electron microscopy.

Three researchers (M.N, R.A., and P.Z.) independently evaluated electron micrographs. Number and width of capillaries were estimated by using the plug-in “cell counter” and the function “measure” in the Image J software (version 1.5)^21^; 3 electron micrographs were evaluated for each donor. Fibrils width and degree of organization of fibrils (anisotropy) were estimated by using the function “measure” and the plug-in FibrilTool^27^ in the Image J software; 3 electron micrographs were evaluated for each donor. Anisotropy was measured on the entire area of electron micrographs, in order to avoid any bias due to the selection of areas excluding or over-representing certain fibers in the outputs. The degree of alignment of fibrils in pair-wised ECMs was estimated on images with the same magnification.

### Statistical analysis and data mining

Three to 10 surgical specimens were used for each investigative technique, and from each specimen paired healthy-perilesional-tumor area were used. Sample size for each investigative technique is reported in Supplementary Material 1. Difference among groups was evaluated by using ANOVA followed by post-test analysis. MeV software (version 4_9_0)^28^ was used for data from proteomic analysis to generate hierarchical clustering heat-map. Data mining was performed using Ingenuity Pathway Analysis (IPA)^29^.

## Results

### Morphology of healthy colon, perilesional area and CRC tissue

Healthy colon mucosa came from the resection margins and was >10 cm from the CRC, whereas the neoplastic tissue was obtained at the edge of the infiltrating neoplasia. The choice of perilesional tissue was based on the lack of epithelial dysplasia, mild architectural abnormalities, and blood vessels with elongated and dilated shape and in a range of 0.5–1 cm from the edge of the neoplastic lesion.

**Figure 1:**
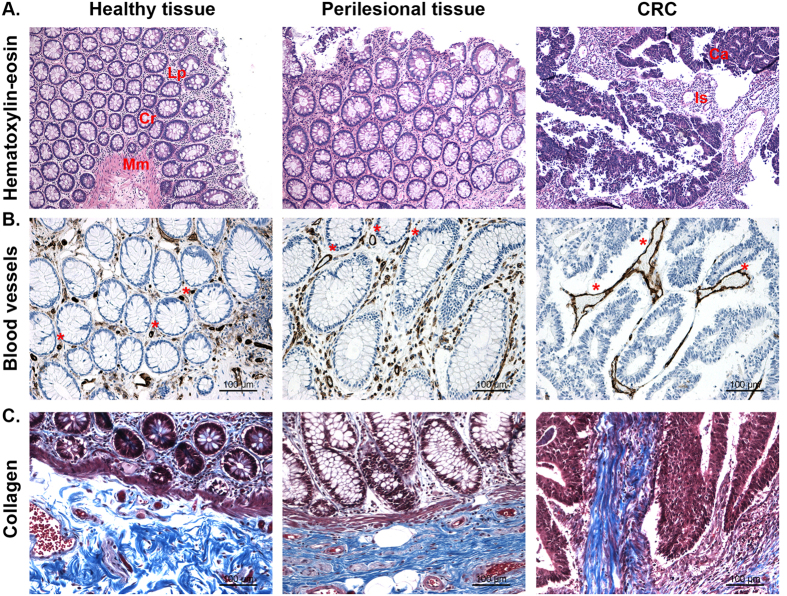
Tissue selection. Pair-wised healthy colon, perilesional area and CRC were evaluated by means of hematoxylin-eosin staining (**A**), CD34+ blood vessels (**B**) and collagen (blue staining) by means of Masson Trichrome stain (**C**). Cr; cryptae. Lp; lamina propria. Mm; muscularis mucosae. Ca; carcinoma. Is; intratumoral stroma. *blood vessels. Pictures are representative of pair-wised tissues from one of the six patients tested and listed in Supplementary Material 1.

Hematoxylin-eosin staining (Fig. 1A), the size and shape of blood vessels (Fig. 1B) and collagen distribution (Fig. 1C) were used to establish morphology of surgical specimens (Supplementary Material 1).

The left colon from the resection margin showed normal architecture, a homogeneous distribution of blood vessels, homogeneous size and distribution of crypts and short wavy bundles of collagen fibres.

Compared to healthy tissue, perilesional mucosa showed mild hyperplastic crypts, increased thickness of the lamina propria and an increased number of blood vessels of larger size (median number of capillaries for 100 crypts was 16 vs 38, and median capillary width of 13 vs 35 μm in the healthy colon mucosa vs peritumoral tissue, Supplementary Fig. S1), associated with straight bundles of collagen fibres in the submucosa. Colorectal carcinoma was characterized by neoplastic glands infiltrating desmoplastic stroma, disorganized architecture, and increased number of enlarged (Supplementary Fig. S1) ectatic vessels and collagen fibres organized in thick straight bundles.

### Differential composition of core matrisome and matrix-associated components in CRC but not perilesional ECM

**Figure 2:**
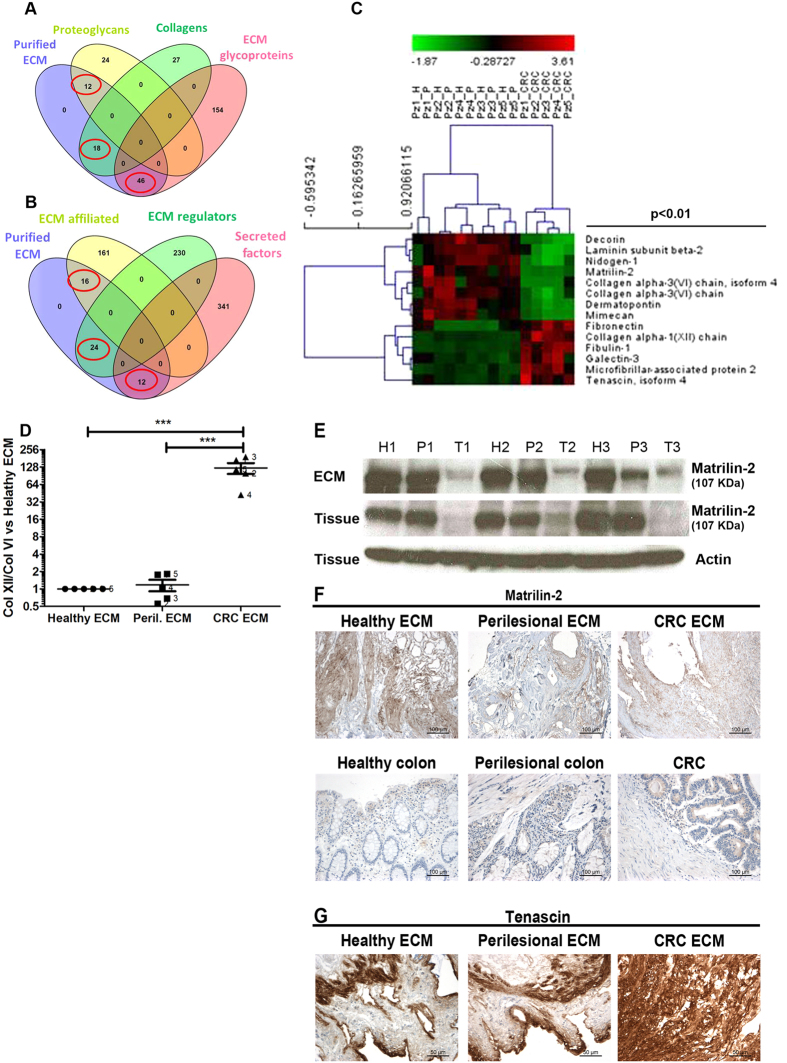
Protein composition of ECMs. ECM was purified from pair-wised colon resection margins (healthy, H), perilesional areas (P) and colorectal carcinomas (CRC) from 5 patients listed in Supplementary Material 1, and proteomic analysis was performed as described in Methods. Comparison of purified ECMs (purple ellipse in the Venn’s diagrams) with the matrisome database^18^ revealed identification of 128 matrisome proteins; 76 structural components (12 proteoglycans, 18 collagens and 46 ECM glycoproteins, as highlighted by red circles in panel (**A**) and 52 matrix-associated components (16 ECM affiliated, 24 ECM regulators and 12 Secreted factors, as highlighted by red circles in panel (**B**) such as i) ECM-affiliated, as annexin, mucin, complement, calcium binding proteins S100; ii) ECM-regulators, as metallo-proteases, metallo-protease inhibitors; iii) Secreted factors bound to ECM, as latent TGF-b, growth factors, chemokines. Unsupervised hierarchical cluster analysis of LFQ intensities as derived from MaxQuant elaboration (Suppl. file 2.1) was performed upon parametric ANOVA test: the heatmap shows significantly differentially expressed ECM proteins with p-value < 0.01 (**C**). The ratio between collagen XII and the sum of the two forms of collagen VI reported in the above heat-map was estimated in all 5 ECMs and fold of expression was calculated vs healthy ECM (**D**). Western blot analysis for Matrilin-2 expression in ECMs and tissues from 3 surgical specimens; level of actin expression in tissue was used as loading control (**E**). Expression of Matrilin-2 was further evaluated by IHC, in pair-wised ECMs (one representative patient is shown) (**F**). Expression of Tenascin was evaluated by IHC (pair-wised tissues from one representative patient are shown) (**G**). (**H**) P and T in panel C and panel E indicate healthy, perilesional and tumor area, respectively. P value in panel C and asterisks in panel D indicate statistical significance.

To assess whether modification of tissue architecture was caused by different protein composition a quantitative proteomic analysis was performed on ECM purified from 5 pairwised tissues obtained from CRC patients (Supplementary Material 1). A total of 1139 non-redundant proteins were measured among the three types of ECMs (Supplementary Material 2.1). Based on the human matrisome database^18^, 128 proteins out of 1139 were ascribed to the human matrisome, with 76 proteins belonging to the core matrisome (Fig. 2A) and 52 proteins being matrix-associated components (Fig. 2B). In the core matrisome group we identified 12 proteoglycans, 18 collagens and 46 glycoproteins out of 36 proteoglycans, 45 collagens and 200 glycoprotiens reported in the human matrisome database (red circles in Fig. 2A, and Supplementary Material 2.2 and 2.3). In the matrix-associated components we identified 16 ECM-affiliated components out of 176, 24 ECM regulators out of 254, and 12 secreted factors out of 353 reported in the human matrisome database (red circles in Fig. 2B and Supplementary Material 2.2 and 2.3).

Unsupervised hierarchical clustering of the 128 ECM proteins showed dysregulation of ECM composition in the CRC-ECM (13 core matrisome and 1 matrix affiliated protein, p < 0.01) vs. the healthy and perilesional ECM, while the healthy vs perilesional ECMs showed no difference (Fig. 2C and Supplementary Material 2.4).

CRC ECM was characterized by a down-modulation of i) basal membrane proteins such as Laminin subunit beta-2 and Nidogen-1, and ii) interstitial matrix proteins (Matrilin-2, full-lenght and isoform 4 of Collagen 6A3, and Mimecan), for some of which (Decorin and Dermatopontin) anti-proliferative activity was previously demonstrated (described in section 4 of Supplementary Material 1). Six interstitial matrix proteins (Fibronectin, Collagen 12A1, Fibulin-1, Galectin-3, Microfibrillar associated protein-2 and Tenascin) were over-expressed in the CRC ECM. A drastic shift in the ratio between collagen 12A1 and collagen 6A3 was observed, with 50–200 fold accumulation of collagen 12 vs collagen 6 in the CRC ECM (Fig. 2D).

To confirm the differential expression of proteins identified by the proteomic analysis, representative down and up modulated proteins such as Matrilin-2 and Tenascin were evaluated on pair-wised samples. Decreased level of Matrilin-2 in CRC, but not in the perilesional area, was confirmed in both ECMs and tissues by western blot (Fig. 2E) and immunohistochemistry (Fig. 2F) analysis. Increased level of Tenascin in CRC was confirmed by immunohistochemistry analysis of ECMs (Fig. 2G).

Other proteins not reported in the matrisome database were also identified in the ECMs. Tumoral antigens (eg, CEACAM5 and OCIAD2) and tumor-associated proteins CD97 were present and overexpressed in the CRC ECM (p < 0.01) and, as for the matrisome proteins, no difference was observed between healthy and perilesional ECMs. In addition, two tumor-associated proteins FAP (fibroblast activation protein/seprase) and DPEP1 (dipeptidase 1) were absent from healthy and perilesional ECM (Supplementary Material 2.5).

An Ingenuity Pathway Analysis (IPA) based on all dysregulated proteins (Supplementary Material 2.6) identified mono-(2-ethylhexyl)phthalate (p = 10^−7^) and beta-estradiol (p = 10^−5^) as the top and activated upstream regulators that could explain the observed differences. The oestrogen pathway was validated on surgical specimens for the expression of estradiol receptor-beta (ER-β). As reported^20^,^30^,^31^, the neoplastic tissue was characterized by i) low numbers of epithelial cells expressing ER-β and ii) low intensity of ER-β, whereas all epithelial cells in the healthy and perilesional tissue were positive for ER-β (Supplementary Fig. S2). All stromal cells expressed ER-β irrespectively of their localization in the healthy tissue, peritumoral area or CRC, with comparable level of ER-β expression (Supplementary Fig. S2).

### Gradient of increasing stiffness in healthy to perilesional and colorectal carcinoma extracellular matrix

**Figure 3:**
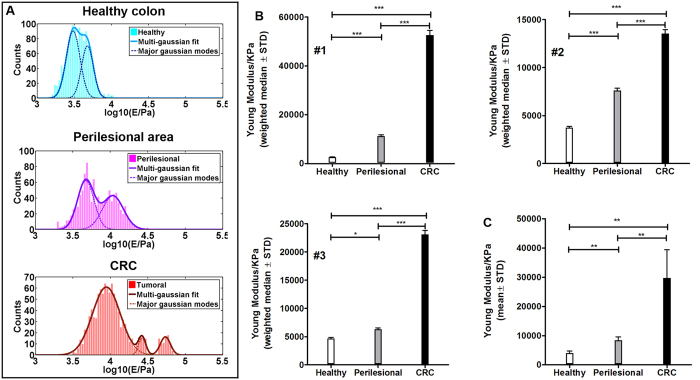
Elastic Young’s modulus of ECM from healthy, perilesional and CRC area. Cumulative distributions of Young’s modulus (E) values in Pa units (semilog10 scale) of the three ECMs derived from 1 representative patient (out of 3 tested and listed in the Supplementary Material 1; multi-gaussian fit representative of the cumulative distribution (solid line) and the contribution of major Gaussian modes (dotted line) were estimated (**A**). Median value for each ECM was estimated by weighting the relative contribution of each mode, and showed for all 3 donors (**B**). Mean value of Young’s modulus of healthy, perilesional and CRC ECM (**C**). Error bars are calculated as standard deviation of the median. Statistical significance was evaluated by means of ANOVA followed by Tukey post-test analysis. E; elasticity. Pa; pascal.

Despite the perilesional area is showing altered architecture and its ECM is susceptible to infiltration by invasive cancer cells^15^, the perilesional ECM components, the amount of FAP bound to the ECM and the number of ER-β+ cells did not differ from the healthy environment. Thus, the physical feature of the ECM was assessed. Stiffness of the ECM derived from the healthy colon, perilesional area and CRC of three donors was measured by atomic force microscopy (AFM) indentation measurements. Broad distributions of the elastic modulus values (Fig. 3A, solid line) was represented by major modes contributing to the cumulative distribution (Fig. 3A, dotted line), suggesting a strong diversity of elastic properties within the same sample. A remarkable overlap of some of the major modes within ECM was always present, although the relative contribution of single modes to the cumulative stiffness was different in the healthy, perilesional and CRC ECM. To take in account the relative contribution of each modes a weighted median was estimated for each ECM (Fig. 3B). Indeed, a common trend was observed for all patients, showing a softer healthy ECM and a stiffer tumoral one, with the perilesional ECM sharing features with both healthy and tumoral cases (Fig. 3C and Supplementary Fig. S3).

The average median value of the Young’s modulus of ECMs from different regions shows similar pattern in each donor. This suggests that, even within the large patient-to-patient and even tissue-to-tissue variability, there are common characteristics related to the physio-pathological state of the tissues that are statistically conserved. Indentation measurements were thus representative of the collective contributions of nanoscale molecular components organized in micrometer-sized structural and functional domains in the ECMs; adjusting the probe diameter^23^,^24^ to the size of these domains allowed detecting and resolving ECMs mechanical responses, monitoring them in relation to the degree of pathology progression.

### Gradient of increasing collagen crosslinking in healthy, perilesional and colorectal carcinoma extracellular matrix

In order to investigate the steady increase of ECM stiffness, independent of protein composition, post-translational modifications of the ECM such as collagen crosslinking was assessed. We measured hydroxylysyl-pyridinoline (HP) and lysyl-pyridinoline (LP) crosslinking, which are formed from oxidative de-amination of hydroxylysine and lysine, respectively, upon the action of the lysyl oxidase (LOX) in the presence of oxygen^32^.

**Figure 4:**
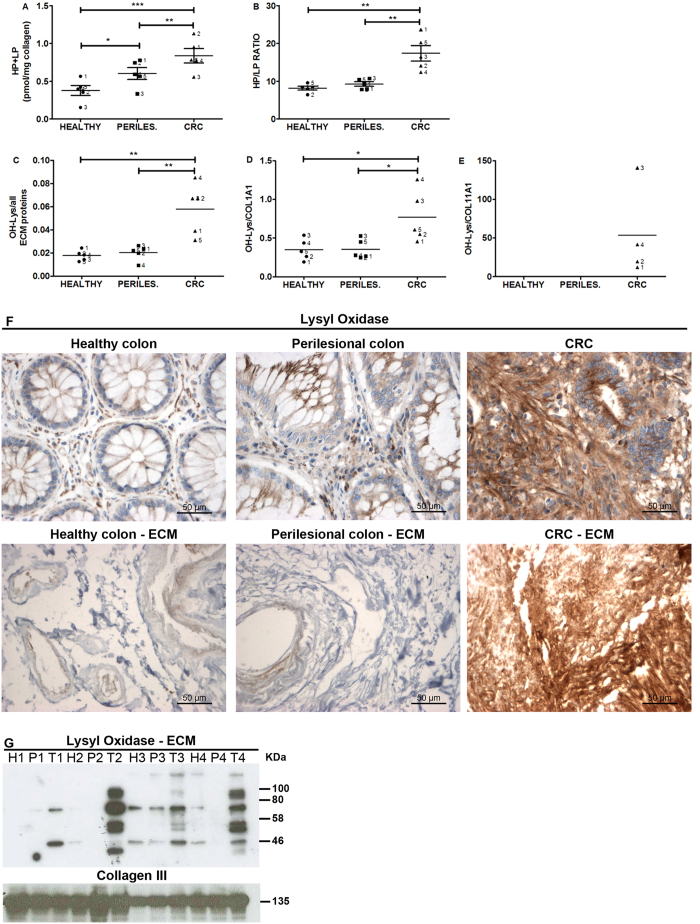
Level of collagen crosslinking, OH-Lys content of ECM proteins and lysyl oxidase in healthy, perilesional area and CRC ECMs. Pair-wised ECMs from tissue of 5 donors (Supplementary Material 1) were evaluated for the content of collagen crosslinking HP and LP (**A**) and the ratio between HP/LP (**B**). Level of OH-lysine was estimated for all ECM proteins: sum of peak intensities, as estimated by MaxQuant software, for all OH-lysine-containing peptides was normalized to sum of ECM protein intensities in each ECM derived from 5 patients (Supplementary Material 2.7) (**C**). Sum of peak intensities, as estimated by MaxQuant software, for all OH-lysine-containing peptides was normalized to each protein intensity in each ECM derived from 5 patients (Supplementary Material 2.8) (**D**, Collagen 1A1; (**E**), Collagen 11A1). Asterisks indicate statistical significance evaluated by means of ANOVA. Immunohistochemistry (40× magnification) of LOX on pair-wised tissues (healthy colon, perilesional area and CRC) and the derived ECMs; data from one representative surgical sample out of 4 tested (**F**). Level of lysyl oxidase was evaluated by western blot using pair-wised ECMs from 4 donors (**G**); the molecular masses (kDa) are indicated on the right side of the panel. H, P and T in panel G indicate that ECM was isolated from pair-wised healthy, perilesional and tumor area, respectively, from 4 different surgical specimens.

An enhanced amount of crosslinking was present in the perilesional vs healthy ECM, and this increase was even more substantial in the CRC ECM (Fig. 4A). The ratio between HP and LP was identical for healthy and perilesional ECM and ca. 2 times greater in CRC (Fig. 4B). Comparable HP/LP ratios in healthy and perilesional ECMs confirmed findings from proteomic analysis, suggesting that both ECMs show similar protein composition, primary structure and post-transcriptional modification. On the other hand, the increased HP/LP ratio in the CRC ECM (Fig. 4B) was associated with an increased level of hydroxylated lysine (Fig. 4C and Supplementary Material 2.7), and among all ECM proteins carrying hydroxylated lysine (Supplementary Material 2.8 and Supplementary Fig. S4) only COL1A1 showed increased content of hydroxylated lysine vs healthy and perilesional ECMs (Fig. 4D). Moreover, COL11A1 with a high level of OH-Lys was expressed only in CRC ECM (Fig. 4E), in agreement with a report showing COL11A1 to be produced by cancer-associated myofibroblasts and not expressed in the healthy colon^33^.

Among collagens, the fibrillar collagen-I is the main substrate for LOX^10^, inducing post-translational modification of hydroxylysine and lysine. To address the increased amount of HP crosslinking with the increased amount of OH-Lysine content in collagen-I, we evaluated distribution and level of expression of LOX. The extracellular enzyme LOX was detected both in the tissues and ECMs by IHC. Distribution and level of expression of LOX was similar between healthy colon and perilesional tissue, and a drastic increase was evident in the CRC; similar profile was evident in the derived ECMs (Fig. 4F). Comparable level of LOX expression between healthy colon and perilesional ECMs, and increased amount of LOX associated to the CRC ECM was also confirmed by western blot analysis (Fig. 4G). This finding was in agreement with previous report^18^. Moreover, different forms of LOX were evident from the western blot analysis, corresponding to the non-glycosylated LOX (molecular weight 47 kDa) and the glycosylated forms (upper bands at 54 and 57 kDa), as already described^34^.

### Ultrastructural analysis of human colon layers ECM

Scanning electron microscopy was used to establish how the biochemical composition and physical modifications were associated with ECM ultrastructure of colon mucosa. The same analysis was further extended to the underlying tissue layers, such as muscularis propria and subserosa.

**Figure 5:**
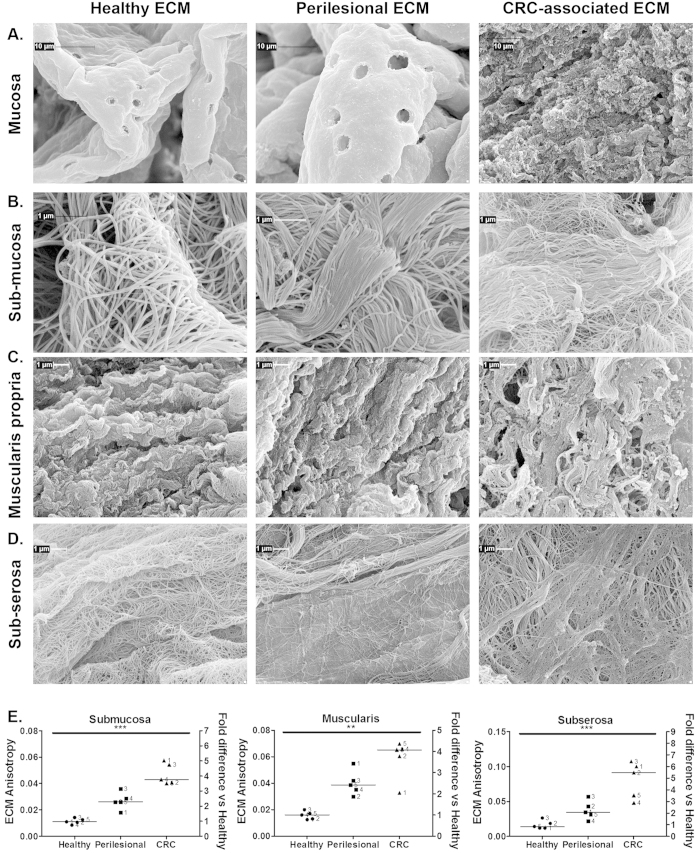
Ultrastructural analysis of ECMs. ECMs from mucosa (**A**), submucosa (**B**), muscularis propria (**C**) and subserosa (**D**) of pair-wised healthy colon, perilesional area and CRC were evaluated by means of scanning electron microscopy. Lower magnifications are reported in the Supplementary Fig. S5. Pictures show ultrastructure of pair-wised ECMs from one representative patient (#2 listed in Supplementary Material 1). Degree of organization of fibrils (anisotropy) was evaluated in pair-wised ECMs from 5 donors (**E**); horizontal bars indicate median value, and asterisks show statistical significance evaluated by means of Anova. Representative scanning electron micrographs used for establishing anisotropy are reported in the Supplementary Fig. S6.

In the ECM derived from healthy mucosa, regular morphology of crypts with homogeneous distribution, honeycomb-like shape and size (50–80 μm width) of empty crypts and homogeneous thickness of the lamina propria between crypts was observed. In the lamina propria of healthy ECM “holes” left from the removal of endothelial cells (i.e., capillaries) were present (Fig. 5A and Supplementary Fig. S5A). Perilesional mucosa ECM showed an irregular topography of lamina propria, highlighting the heterogeneous shape and diameter of crypts (Fig. 5A and Supplementary Fig. S5A). In addition, the lamina propria of perilesional ECM was characterized by an increased number of capillaries (262 ± 14 vs. 146 ± 16 per mm^2^, ECMs from 3 different surgical specimens, p = 0.002) and increased capillary width (5.3 ± 0.1 vs. 2.9 ± 0.05 μm, n = 3 for a total of 146 capillaries, p = 0.0001) (Fig. 5A and Supplementary Fig. S5A) compared to healthy ECM, with a fold of increase of 1.8 in agreement with the fold of increase observed by immunohistochemical analysis of tissues (Supplementary Fig. S1). Mucosa associated with CRC ECM was characterized by an irregular and indistinct morphology (Fig. 5A and Supplementary Fig. S5A); dilated/ectatic vessels in the CRC ECM were not evaluated because not distinguishable from empty spaces present in the neoplastic stroma.

In the submucosa of healthy and perilesional ECM, morphology with a wavy pattern was observed (Fig. 5B and Supplementary Fig. S5B). Both healthy and perilesional ECMs were constituted by thousands of fibers, though organization and thickness were very different. Fibrils in healthy ECM (Fig. 5B) mainly intersected each other to form a random network. The random network of relaxed and crosslinked fibrils in the healthy ECM become progressively linearized in the perilesional ECM, forming tight and organized in bundles (Fig. 5B). Submucosa ECM associated with CRC was characterized by a loss of tissue morphology, as reported for human adenocarcinoma^35^, with fibrils organized in tight bundles and thinner than that observed in the healthy and perilesional ECM (Fig. 5B and Supplementary Fig. S5B) (fibrils width; 40 ± 8 vs 90 ± 26 nm, p < 0.0001, 52 measurements in pair-wised ECMs from 3 donors).

The ECM from the healthy colon muscularis propria showed a structure with a regular morphology of crests and clefts and a well-oriented direction of fibers constituted by parallel fibrils organized in intertwisted bundles (Fig. 5C and Supplementary Fig. S5C). Crests and clefts were less evident in the perilesional muscularis propria ECM due to fibrils being condensed in a denser matrix (Fig. 5C and Supplementary Fig. S5C). Loss of organized bundles and architecture was evident in the CRC-associated muscularis propria ECM, with the fibrils organized to form a heterogeneous structure characterized by irregular spaces (Fig. 5C and Supplementary Fig. S5C).

The healthy subserosa ECM was composed of many homogeneous and well-separated fibrils forming a mesh with regular spaces in the network (Fig. 5D and Supplementary Fig. S5D). In the perilesional subserosa ECM fibrils were of the same width as in the healthy ECM (60 ± 0.9 vs 62 ± 1.3 nm) but were linearized and mainly organized in bundles (Fig. 5D and Supplementary Fig. S5D). In the CRC-associated subserosa ECM fibrils were thinner (55 ± 1.2 nm, p < 0.001 vs healthy and perilesional), linearized and mainly organized to form a mesh of heterogeneous structure, characterized by irregular spaces among the bundles (Fig. 5D and Supplementary Fig. S5D).

The ultrastructural modifications were quantified by assessing the degree of alignment of fibrils (anisotropy). Increased anisotropy was observed in both perilesional and CRC ECMs vs healthy ECM, and the gradient was common to the submucosa, muscularis propria and subserosa (Fig. 5E).

## Discussion

We recently reported that perilesional, but not healthy, ECM supports cell invasion^15^. This study provides an in-depth investigation of both physical and biochemical features of healthy, perilesional and neoplastic ECMs and identifies stiffness and crosslinking of the perilesional ECM as the main parameters associated with infiltration of cancer cells (Table 1). In agreement with the gradient of stiffness and amount of crosslinking from healthy to perilesional and CRC area, we also report a gradient in the modification of the ultrastructure characterized by increased degree of fibril organization and disorganized architecture common to all colon tissue layers. Moreover, the broad distribution of the elastic modulus reflected ultrastructural changes in the perilesional and CRC areas indicating that nanomechanical measurements using colloidal probes^22^,^23^ may potentially be a predictive tool for assessing the modification of ultrastructural changes associated with CRC progression.

In the CRC we confirmed the modulation of the oestrogen pathway at the level of neoplastic cells and the presence of a poorly organized vascular network^35^ composed by ectatic vessels, previously reported to be associated with a hypoxic condition^35^,^36^,^37^. Analysis of ECM protein content confirmed the invasive pattern of CRC, as previously described on tissues (down-modulated decorin^38^,^39^, nidogen-1^40^, laminin subunit beta-2^40^,^41^,^42^, dermatopontin^43^, mimecan^44^ and up-modulated fibronectin^45^, tenascin^46^ and collagen 12^9^,^47^).

Lysyl oxidase (LOX) in the presence of oxygen catalyzes an oxidative de-amination of lysine and hydroxylysine residues, spontaneously condensing to form LP and HP crosslinks in collagen and elastin^32^. In line with our data, the increased stiffness of tissues has previously been correlated with an enhanced i) amount of lysine-derived crosslinks and ii) HP/LP ratio^48^,^49^,^50^, and an increased level of HP was associated with irreversible fibrosis^51^. We also detected an increased level of LOX in the CRC ECM, previously reported to be enhanced in hypoxic environments^52^,^53^. Concomitantly the CRC ECM was also highly enriched in LOX substrate^10^ as revealed by the increased amount of OH-lysine content in in COL1A1.

Neoplasia-associated ECM was deeply re-arranged in terms of both biochemical and physical features. The physical and biochemical diversity of CRC ECM was evident from the formation of bundles composed by linearized and organized fibrils, the increased stiffness and number of collagen crosslinking, change in the matrisome composition and an increased hydroxylation of lysine. In this study we associate the modified ultrastructure and increased stiffness of CRC desmoplastic stroma to the i) enhanced amount of OH-Lysine-derived hydroxylysyl-pyridinoline (HP) crosslinking, and ii) dys-regulated composition of ECM.

The perilesional area of CRC has been described as a histologically normal appearing epithelium with elongated crypts, with thicker lamina propria than normal mucosa and the presence of vessels with elongated and dilated shape^35^.

We confirm the increased vascular density and wider capillary diameter in the perilesional mucosa, despite the fact that tumor-associated pathways were absent in the perilesional ECM (eg, decreased number of ER-β+ epithelial cells, dysmodulation of ECM proteins, CEACAM5, OCIAD2 and FAP bound to neoplastic ECM). Compared to the healthy ECM, perilesional ECM was characterized by increased vascularization, linearized fibrils organized in bundles and increased crosslinking and stiffness, and those modifications were independent of modified protein composition.

Despite the unchanged level of collagens, OH-Lysine content in ECM proteins and LOX, the amount of crosslinking in the perilesional ECM was higher than in normal ECM and its ultrastructure recapitulated the neoplastic ECM. LOXs’ activity depends on the amount of substrates as collagen/elastin and molecular oxygen, suggesting that the increased vascularization/oxygenation of the perilesional area likely represents the responsible factor for the increased crosslinking and stiffness of perilesional ECM.

Moreover, we provide findings here indicative of different mechanisms sustaining an increased level of collagen crosslinking and stiffness of the perilesional and CRC ECMs. The desmoplastic tumoral stroma is composed by dysregulated composition of ECM proteins, with a change in the ratio among collagen XII/VI, increased HP crosslinking consequent to enhanced hydroxylation of lysine in COL1A1. In the perilesional area the desmoplastic stroma is independent of altered i) composition of ECM, ii) level of LOX or iii) OH-lysine level in the ECM protein. The increased vascularization/oxygenation of the perilesional area accounts for the increased HP and LP levels in the perilesional desmoplastic stroma.

Increased crosslinking, stiffness and differently organized ECM fibrils of the perilesional area provide an extracellular environment suitable for tumor invasion and progression. These findings underlie two mechanisms in the CRC pathogenesis. First, cancer cells instruct surrounding tissues to undergo changes that promote malignancy, for example by endorsing neo-angiogenesis or mechanically-induced over-expression of proteins involved in epithelial cell-cell adhesion and gene transcription, as β-catenin^54^. Indeed, mechanical loading has also recently been reported to increase LOX-mediated crosslinking^55^. Modifications in the vascularized and stiffer perilesional area might represent a feed-forward loop which spreads features of the neoplastic ECM.

Second, our results also suggest that mechanical pre-strain of the ECM might predispose a soil needed for the onset of CRC. We observed that in the perilesional area the level of LOX was unchanged vs the healthy tissue. The pre-strain of perilesional ECM is likely supported by the enhanced vascularization of the tissue, providing increased amount of oxygen which is one of the substrate of the enzymatic reaction mediated by LOX. This interpretation is supported by epidemiological studies reporting that patients with Crohn’s disease are characterized by enhanced intestinal vascularization and matrix stiffness and experience an increased incidence of CRC^56^. Similarly, a study from an animal model showed that microvascular blood content increased very early in colon carcinogenesis prior to the development of adenomas^57^.

These findings provide a framework for alternative therapeutic strategies against CRC progression, as chemoresistance may be overcome by targeting the tumor surrounding microenvironment^58^ and level of pyridinoline crosslinking might be controlled by anti-oxidant agents^59^, anti-fibrotic agents^60^ or through normalization of vasculature^36^.

This study identifies stiffness and crosslinking of the ECM as the main parameters responsible for the “soil effect” of the perilesional area, such as increased proliferation and turn-over of cancer cells and permissiveness to infiltration^15^. Furthermore, relative increased stiffness of colon areas, within the same patient, may represent a new predictive marker of desmoplastic region, predisposing to invasion, thus offering new potential application for monitoring adenoma with invasive potential.

## Additional Information

**How to cite this article**: Nebuloni, M. *et al*. Insight On Colorectal Carcinoma Infiltration by Studying Perilesional Extracellular Matrix. *Sci. Rep.* **6**, 22522; doi: 10.1038/srep22522 (2016).

## Supplementary Material

Supplementary Material 1

Supplementary Material 2

## Figures and Tables

**Table 1:** Modification of the features of perilesional and CRC ECMs vs healthy ECM.

	**PERILESIONAL**	**CRC**
Morphology	Irregular	Loss of morphology
Ultrastructure	Organized and linearized	Fibrils fused in tight bundles
Anisotropy	Increased (2×)	Increased (4×)
Vascularization	Increased (2×)	Ectatic vessels
Stiffness	Increased (2.5×)	Increased (9.4×)
Proteomic	No difference	Different, and increased OH-Lys
Crosslinking	Increased (1.5×)	Increased (2×), with 2× increased HP/LP ratio
Cell proliferation^15^	Increased	Increased
Cell apoptosis^15^	As in healthy	Delayed
Invasion^15^	Present	Present

Fold difference vs healthy ECM was calculated using median values. Cell proliferation, apoptosis and invasion by metastatic cells seeded on healthy colon, perilesional and CRC derived ECM was recently reported^15^.
