# Supplementary material for: Insight On Colorectal Carcinoma Infiltration by Studying Perilesional Extracellular Matrix
Source: Sci Rep. 2016 Mar 4;6:22522. doi: 10.1038/srep22522 (PMC4778019; doi:10.1038/srep22522)
Supplement: Supplementary Material 1 [file srep22522-s1.pdf]

# INSIGHT ON COLORECTAL CARCINOMA INFILTRATION BY STUDYING PERILESIONAL EXTRACELLULAR MATRIX

**List of investigators:** Manuela Nebuloni, Luca Albarello, Annapaola Andolfo, Cinzia Magagnotti, Luca Genovese, Irene Locatelli, Giovanni Tonon, Erika Longhi, Pietro Zerbi, Raffaele Allevi, Alessandro Podestà, Luca Puricelli, Paolo Milani, Armando Soldarini, Andrea Salonia, Massimo Alfano.

## ADDITIONAL INFORMATION

**Table of contents:**

1. Supplementary Figures
2. Supplementary Table S1. Clinico-pathological features of left colon cancer patients and investigative techniques used on each surgical sample.
3. Supplementary Figure Legends
4. Significance of down and up regulated proteins in CRC ECM
5. References

# 1. SUPPLEMENTARY FIGURES

SUPPLEMENTARY FIGURE S1

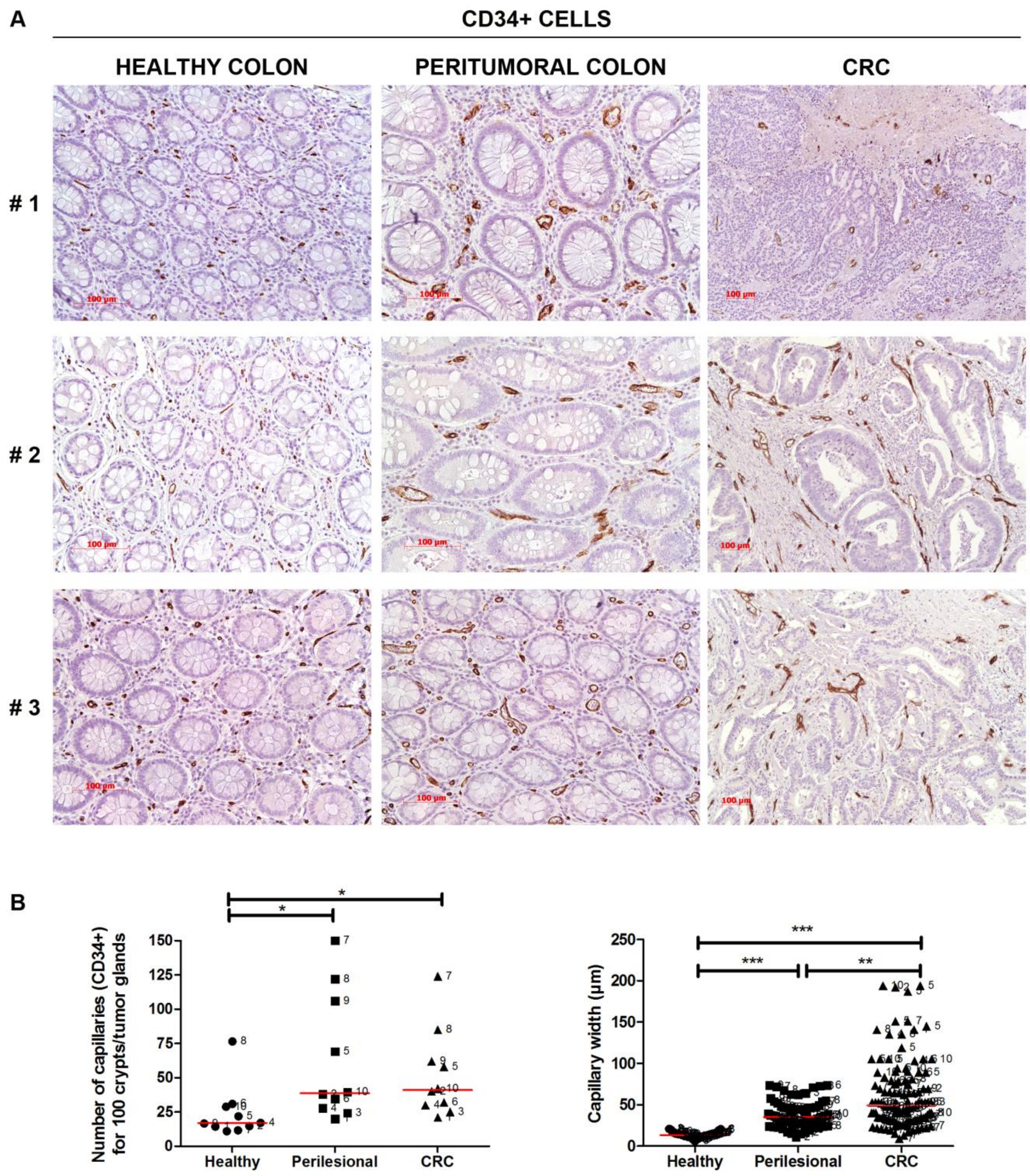

**SUPPLEMENTARY FIGURE S2**

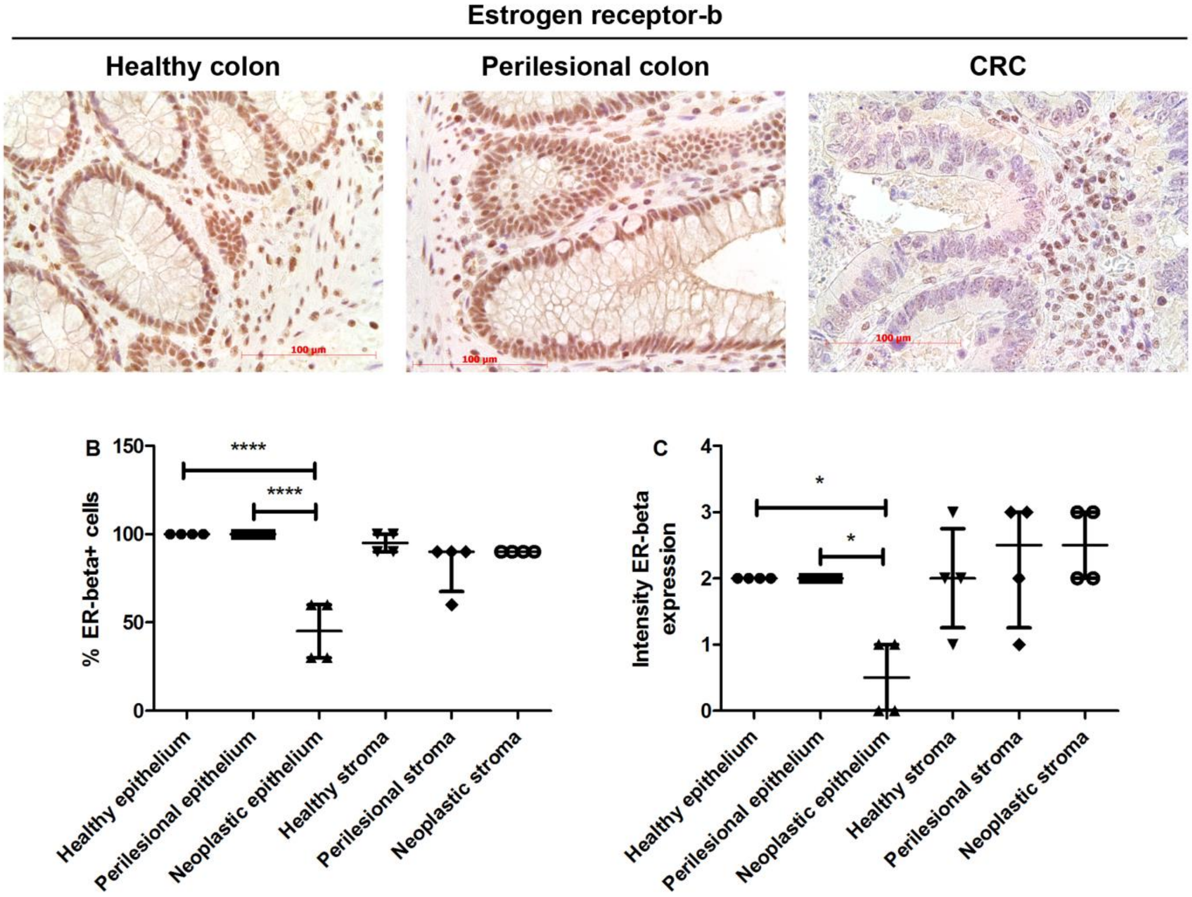

**SUPPLEMENTARY FIGURE S3**

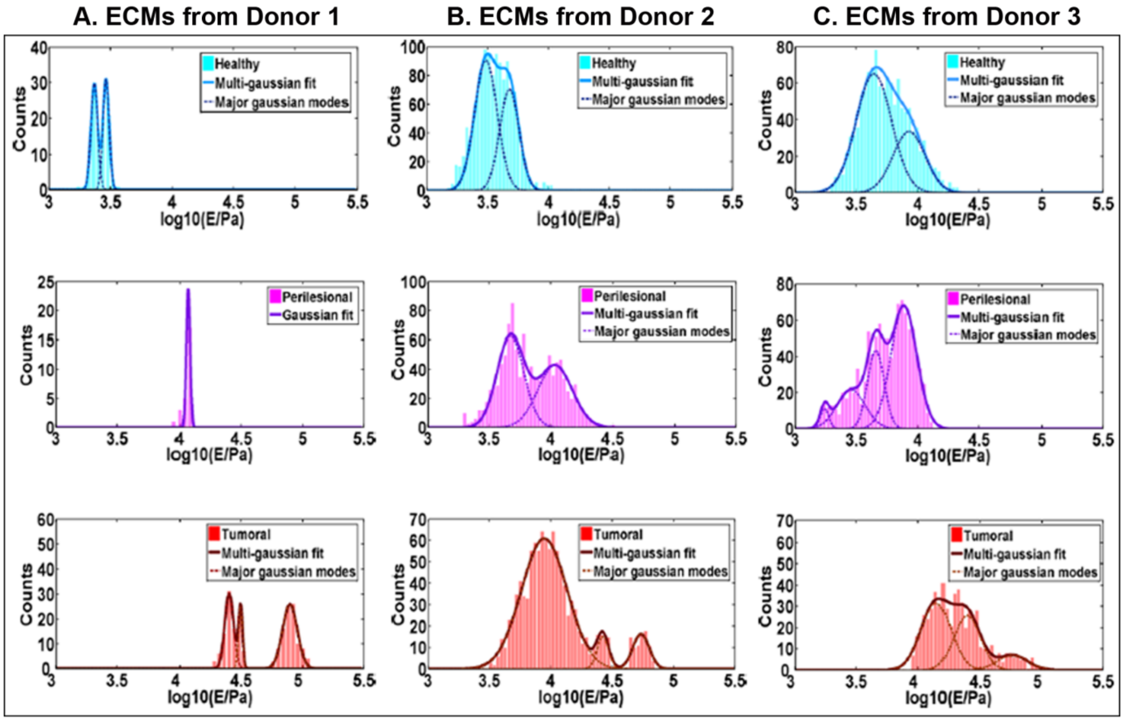

## SUPPLEMENTARY FIGURE S4

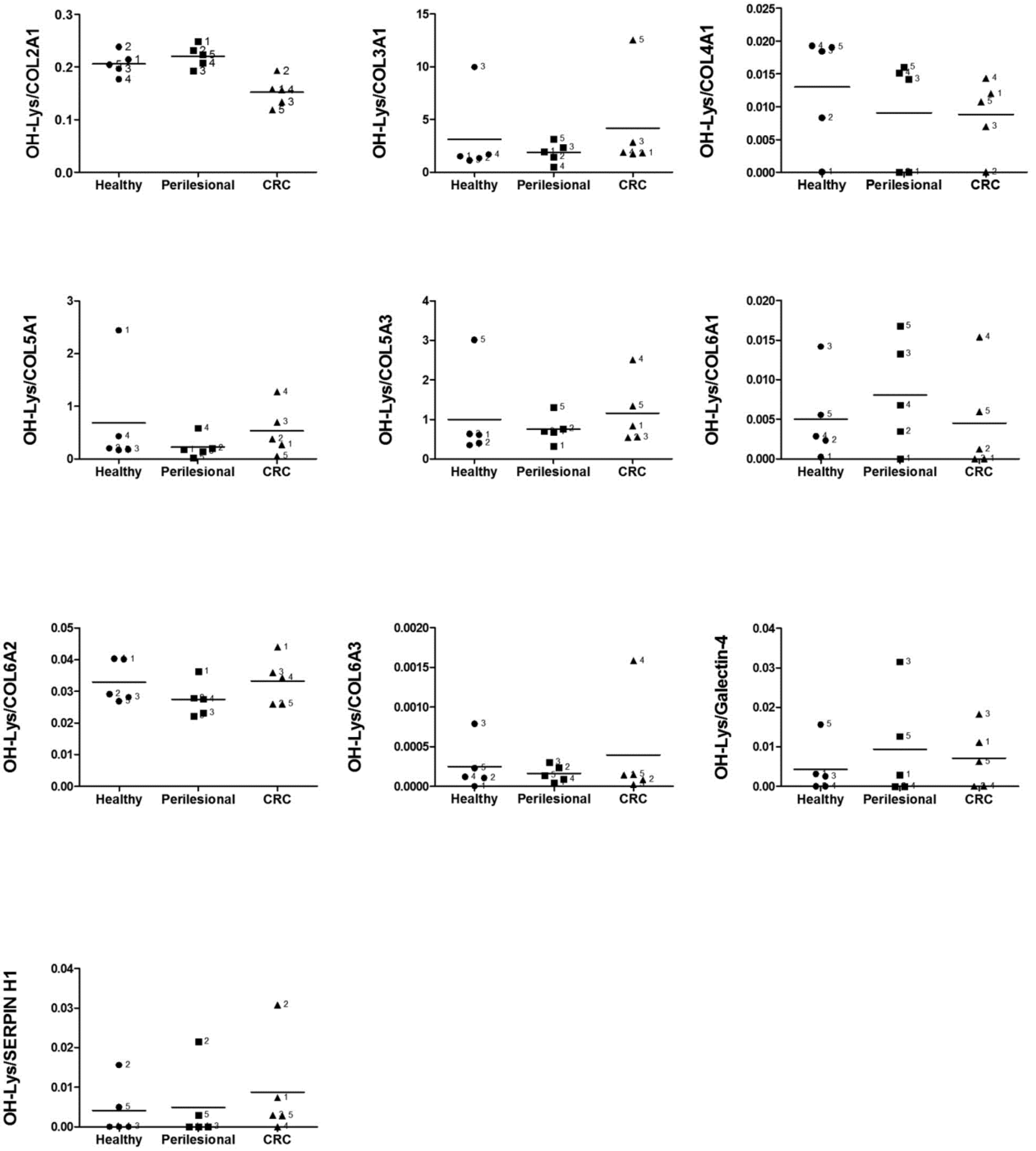

**SUPPLEMENTARY FIGURE S5**

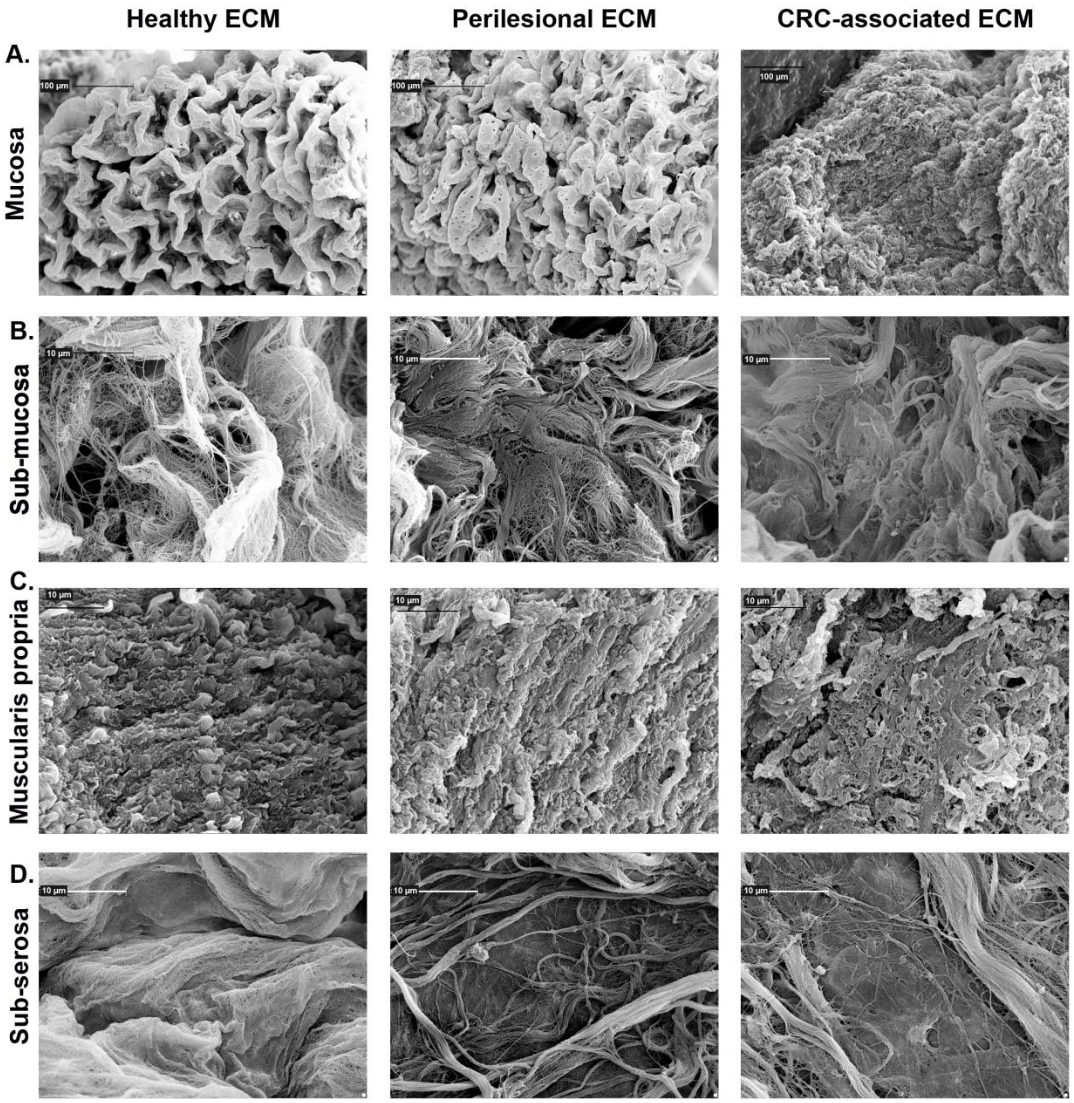

**Supplementary Figure S6**

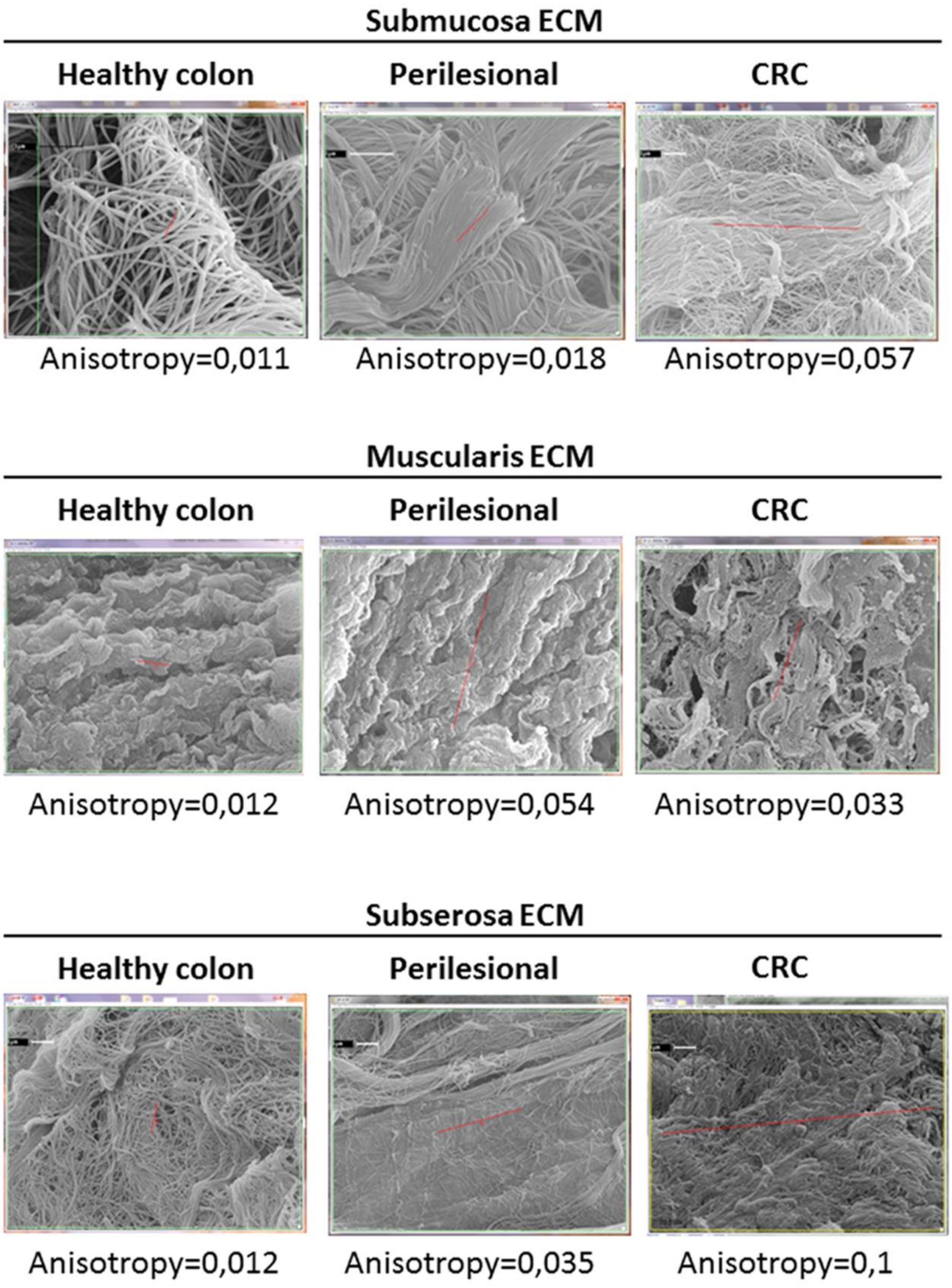

**2. Supplementary Table S1. Clinico-pathological features of left colon cancer patients and investigative techniques used on each surgical sample.**

| #[a] | Age | Sex[b] | TNM, Grade[c] | Morphology and structural study | CD34+ cells | Proteomic analysis | Matrilin-2, Tenascin Validation | ER-b | Stiffness | Crosslinking | LOX analysis |
|---|---|---|---|---|---|---|---|---|---|---|---|
| **1** | 75 | F | pT2N0, G2 | x | | | | | | | |
| **2** | 74 | M | pT3N1a, G2 | x | x | | | | | | |
| **3** | 71 | F | pT2N1a, G2 | x | x | | | | | | |
| **4** | 74 | M | pT3N0, G2 | x | x | | | | | | |
| **5** | 82 | M | pT3N0, G2 | x | x | | | | | | |
| **6** | 77 | F | pT3N1b, G3 | x | x | | | | | | |
| **7** | 78 | M | pT4aN1b, G2 | | x | | | | | | |
| **8** | 58 | M | pT3N0, G2 | | x | | | | x | x | x |
| **9** | 79 | M | pT3N0, G3 | | x | x | x | x | | | |
| **10** | 68 | M | pT3N0, G3 | | x | x | x | x | | | |
| **11** | 55 | F | pT3N0, G2 | | x | x | x | x | | | |
| **12** | 72 | F | pT3N1a, G2 | | | x | x | x | | | |
| **13** | 66 | M | pT3N0, G3 | | | x | x | | | | |
| **14** | 92 | M | pT4aN2a, G3 | | | | | | | x | |
| **15** | 73 | F | pT3N0, G2 | | | | | | x | x | |
| **16** | 63 | F | pT3N0, G2 | | | | | | x | x | |
| **17** | 71 | M | pT3N1a, G2 | | | | | | | x | |
| **18** | 65 | M | pT3N0, G2 | | | | | | | | x |
| **19** | 76 | M | pT3N1b, G2 | | | | | | | | x |
| **20** | 68 | M | pT4aN1a, G2 | | | | | | | | x |

[a]Anonymized code. [b]Male/Female. [c]TNM classification ($7^{th}$ Edition, 2009).

## 3. SUPPLEMENTARY FIGURE LEGENDS

**Supplementary Figure S1. Increased number and capillary width in the perilesional colon mucosa.** Ten pair-wised tissues were investigated for the level of CD34+ cells (Supplementary Table S1). Representative pair-wised pictures from three donors are shown (A). Number of capillaries was evaluated on all 10 pair-wised tissues and expressed for 100 crypts or neoplastic glands, and capillary width estimated on all counted capillaries (B); median values are shown by red bar. Statistical significance, indicated by asterisks, was evaluated by two-tailed paired t-test. Selection of capillaries was based on CD34+ staining and presence of lumen. Healthy and perilesional tissues are shown at 20x magnification, whereas CRC at 10x magnification.

**Supplementary Figure S2. Down-modulation of ER-b receptor in neoplastic epithelial cells.** The oestrogen pathway identified through IPA, based on all differentially expressed ECM proteins, was validated by means of immunohistochemistry for estrogen receptor-b on healthy, perilesional and CRC tissue (A). Number of ER-b+ cells (B) and levels of ER-b expression (C) in pair-wised healthy colon, perilesional area and CRC were evaluated on pair-wised tissues from 4 patients (representative images are from pair-wised tissues of patient #9 listed in Supplementary Table S1). Intensity of ER-b expression was evaluated on a scale from 0 to 3.

**Supplementary Figure S3. Elastic Young's modulus of ECM from healthy, perilesional and CRC area.** The cumulative distributions of Young's modulus values of ECMs derived from 3 patients listed in Supplementary Table S1 were assessed. From distributions of Young's modulus values in semilog10 scale (A-C, bold line), the median values of the broad distributions as well as of their major modes (A-C, dotted lines) were estimated.

**Supplementary Figure S4. Level of OH-lysine in ECM proteins.** Sum of peak intensities was estimated by MaxQuant software for all OH-lysine-containing peptides, and normalized to each protein intensity in each ECM derived from 5 patients.

**Supplementary Figure S5. Ultrastructural analysis of ECMs.** Lower magnification of pictures reported in Figure 5, representing broader field of ECMs. Pictures from representative pair-wised ECMs are shown.

**Supplementary Figure S6. Anisotropy of ECMs.** The degree of organization of fibrils was established by means of the plug-in FibriTool in the ImageJ software. The output from FibrilTool is represented by a red line, the angle of which represents the average orientation and the length of which is proportional to the array of anisotropy. Representative images are shown. The colored box (green or yellow) within the electron micrographs represents the area selected for the measure,

## 4. Significance of down and up regulated proteins in CRC ECM

### Down-regulated proteins in CRC.

**Decorin:** Connective tissue, interstitial matrix. Extracellular proteoglycan which acts as an important regulator of collagen fibrillogenesis, and inhibitor of cellular proliferation[1] via sequestration of TGF-b and other growth factors[2]. Loss of Decorin has been associated with tumor aggressiveness and unfavorable prognosis[3,4] in urothelial bladder cancer[5].

**Laminin subunit beta 2:** Laminins, a family of extracellular matrix glycoproteins, are the major noncollagenous constituent of basement membranes. They have been implicated in a wide variety of biological processes including cell adhesion, differentiation, migration, signaling, neurite outgrowth and metastasis (http://www.ncbi.nlm.nih.gov/gene?Db=gene&Cmd=ShowDetailView&TermToSearch=3913).

**Nidogen 1:** *Nidogen*-1/entactin-1[6] and *nidogen*-2/entactin-2[7] are basement membrane (BM) glycoproteins mainly expressed by mesenchymal cells and deposited into the epithelial and endothelial BMs during development[8]. Nidogen-1 and nidogen-2 have similar structures and abilities to bind to ECM proteins. Nidogens are reported to be highly sensitive to proteolytic cleavage, although the binding of nidogen-1 to laminin-γ1 decreases susceptibility to proteolysis [9]. Similarly, the binding of nidogen-1 to laminin-γ1 also protects laminins from proteolysis, therefore contributing to BM stability, whereas the removal of nidogens contributes to BM disintegration, thus favoring epithelial-mesenchymal transition and metastasis[8].

**Matrilin 2:** This gene encodes a member of the von Willebrand factor A domain containing protein family. This family of proteins is thought to be involved in the formation of filamentous networks in the extracellular matrices of various tissues. This protein contains five von Willebrand factor A domains. The specific function of this gene has not yet been determined. (http://www.ncbi.nlm.nih.gov/gene/4147). Lack of Matrilin-2 Favors Liver Tumor Development via Erk1/2 and GSK-3β Pathways i*n Vivo*[10].

**Col 6A3 and isoform 4:** Connective tissue, interstitial matrix. The alpha-3 chain is one of the three alpha chains of type VI collagen, a beaded filament collagen found in most connective tissues. The alpha-3 chain of type VI collagen is much larger than the alpha-1 and -2 chains. This difference in size is largely due to an increase in the number of subdomains, similar to von Willebrand Factor type A domains, that are found in the amino terminal globular domain of all the alpha chains. These domains have been shown to bind extracellular matrix proteins, an interaction that explains the importance of this collagen in organizing matrix components. (http://www.ncbi.nlm.nih.gov/gene?Db=gene&Cmd=ShowDetailView&TermToSearch=1293). Growth Promoting Activities of Collagen 6A3[11].

**Dermatopontin:** Seems to mediate adhesion by cell surface integrin binding. May serve as a communication link between the dermal fibroblast cell surface and its extracellular matrix environment. Enhances TGFB1 activity. Inhibits cell proliferation. Accelerates collagen fibril formation, and stabilizes collagen fibrils against low-temperature dissociation (http://www.uniprot.org/uniprot/Q07507). Down-regulated in colorectal adenoma and CRC[12].

**Mimecan:** Induces bone formation in conjunction with TGF-beta-1 or TGF-beta-2. This protein is a small proteoglycan which contains tandem leucine-rich repeats (LRR). Gene ontology annotations related to this gene include growth factor activity (http://www.uniprot.org/uniprot/P20774). Down-regulated in colorectal adenoma and CRC[13].

**Up-regulated proteins in CRC.**

**Fibronectin:** A multifunctional and adhesive glycoprotein widely distributed in connective tissues and subendothelial matrices, as well as in many cell types. Fibronectin is present in a soluble form in body fluids and in an insoluble form in the ECM where it interacts with many other matrix components as well as collagen, fibrin, several integrins and syndecans[14]. Fibronectin originates from a primary transcript, which can be alternatively spliced generating at least 20 different variants.

Fibronectins are involved in cell adhesion, cell motility, opsonization, wound healing, and maintenance of cell shape. Involved in osteoblast compaction through the fibronectin fibrillogenesis cell-mediated matrix assembly process, essential for osteoblast mineralization. Anastellin binds fibronectin and induces fibril formation. This fibronectin polymer, named superfibronectin, exhibits enhanced adhesive properties. Both anastellin and superfibronectin inhibit tumor growth, angiogenesis and metastasis (http://www.uniprot.org/uniprot/P02751).

Fibronectin has been implicated in carcinoma development[15]. In lung carcinoma, fibronectin expression is increased, especially in non-small cell lung carcinoma. The adhesion of lung carcinoma cells to fibronectin enhances tumorigenicity and confers resistance to apoptosis-inducing chemotherapeutic agents. Fibronectin has been shown to stimulate the gonadal steroids that interact with vertebrate androgen receptors, which are capable of controlling the expression of cyclin D and related genes involved in cell cycle control. These observations suggest that fibronectin may promote lung tumor

growth/survival and resistance to therapy, and it could represent a novel target for the development of new anticancer drugs.

**Collagen 12A1:** Connective tissue, interstitial matrix. The alpha chain of type XII collagen, a member of the FACIT (fibril-associated collagens with interrupted triple helices) collagen family. Type XII collagen is a homotrimer found in association with type I collagen, an association that is thought to modify the interactions between collagen I fibrils and the surrounding matrix (http://www.uniprot.org/uniprot/Q99715). Colon adenoma-adenocarcinoma progression is associated with overexpression of collagen XII[16].

**Fibulin 1:** Incorporated into fibronectin-containing matrix fibers. May play a role in cell adhesion and migration along protein fibers within the extracellular matrix (ECM). Homomultimerizes and interacts with various extracellular matrix components such as FN1, LAMA1, LAMA2, and contribute to the supramolecular organization of ECM architecture, in particular to those of basement membranes through its binding to laminin, nidogen and collagen IV. Widely expressed during embryonic development. Four splice variants which differ in the 3' end have been identified. Each variant encodes a different isoform, but no functional distinctions have been identified among the four variants (http://www.genecards.org/cgi-bin/carddisp.pl?gene=FBLN1).

Has been implicated in a role in cellular transformation and tumor invasion, it appears to be a tumor suppressor. May play a role in haemostasis and thrombosis owing to its ability to bind fibrinogen and incorporate into clots (http://www.uniprot.org/uniprot/P23142).

**Galectin 3:** This gene encodes a member of the galectin family of carbohydrate binding proteins. Members of this protein family have an affinity for beta-galactosides. The encoded protein is characterized by an N-terminal proline-rich tandem repeat domain and a single C-terminal

carbohydrate recognition domain. This protein can self-associate through the N-terminal domain allowing it to bind to multivalent saccharide ligands. This protein localizes to the extracellular matrix, the cytoplasm and the nucleus. This protein plays a role in numerous cellular functions including apoptosis, innate immunity, cell adhesion and T-cell regulation. Alternate splicing results in multiple transcript variants (http://www.genecards.org/cgi-bin/carddisp.pl?gene=LGALS3).
Galectin-3 is a galactose-specific lectin which binds IgE. May mediate with the alpha-3, beta-1 integrin the stimulation by CSPG4 of endothelial cells migration. Together with DMBT1, required for terminal differentiation of columnar epithelial cells during early embryogenesis (By similarity). In the nucleus: acts as a pre-mRNA splicing factor. Involved in acute inflammatory responses including neutrophil activation and adhesion, chemoattraction of monocytes macrophages, opsonization of apoptotic neutrophils, and activation of mast cells (http://www.uniprot.org/uniprot/P17931#section_comments).

**Microfibrillar associated protein 2:** Microfibrillar-associated protein 2 is a major component of elastin-associated microfibrils and a candidate for involvement in the etiology of inherited connective tissue diseases. Four transcript variants encoding two different isoforms have been found for this gene. GO annotations related to this gene include *fibronectin binding* and *fibrinogen binding*. An important paralog of this gene is MFAP5 (http://www.genecards.org/cgi-bin/carddisp.pl?gene=MFAP2).
Through $\alpha_V\beta_3$ integrin-mediated signaling, MAGP2 promotes tumor and endothelial cell survival, and its overexpression associated with poor prognosis in ovarian cancer patients[17].

**Tenascin:** Extracellular matrix protein with a spatially and temporally restricted tissue distribution. This protein is homohexameric with disulfide-linked subunits, and contains multiple EGF-like and fibronectin type-III domains (http://www.genecards.org/cgi-bin/carddisp.pl?gene=TNC). TNC is as an extracellular matrix protein of stem cell niches and component of the metastatic niche. Cancer cell–

derived TNC promotes the survival and outgrowth of pulmonary micrometastases, until the tumor stroma takes over as a source of TNC[18].

## 5. REFERENCES

1. Stander, M., Naumann, U., Wick, W. & Weller, M. Transforming growth factor-beta and p-21: multiple molecular targets of decorin-mediated suppression of neoplastic growth. *Cell Tissue Res* **296**, 221-227 (1999).
2. Neill, T., Schaefer, L. & Iozzo, R.V. Decorin: a guardian from the matrix. *Am J Pathol* **181**, 380-387 (2012).
3. Iozzo, R.V., *et al.* Decorin antagonizes IGF receptor I (IGF-IR) function by interfering with IGF-IR activity and attenuating downstream signaling. *J Biol Chem* **286**, 34712-34721.
4. Niedworok, C., *et al.* Inhibitory role of the small leucine-rich proteoglycan biglycan in bladder cancer. *PLoS One* **8**, e80084.
5. Sainio, A., *et al.* Lack of decorin expression by human bladder cancer cells offers new tools in the therapy of urothelial malignancies. *PLoS One* **8**, e76190.
6. Timpl, R., Dziadek, M., Fujiwara, S., Nowack, H. & Wick, G. Nidogen: a new, self-aggregating basement membrane protein. *Eur J Biochem* **137**, 455-465 (1983).
7. Kohfeldt, E., Sasaki, T., Gohring, W. & Timpl, R. Nidogen-2: a new basement membrane protein with diverse binding properties. *J Mol Biol* **282**, 99-109 (1998).
8. Dziadek, M. Role of laminin-nidogen complexes in basement membrane formation during embryonic development. *Experientia* **51**, 901-913 (1995).
9. Mayer, U., *et al.* Binding properties and protease stability of recombinant human nidogen. *Eur J Biochem* **227**, 681-686 (1995).
10. Fullar, A., *et al.* Lack of Matrilin-2 favors liver tumor development via Erk1/2 and GSK-3beta pathways in vivo. *PLoS One* **9**, e93469 (2014).

11. Martianov, I., *et al.* TAF4 inactivation reveals the 3 dimensional growth promoting activities of collagen 6A3. *PLoS One* **9**, e87365 (2014).

12. Pesson, M., *et al.* A gene expression and pre-mRNA splicing signature that marks the adenoma-adenocarcinoma progression in colorectal cancer. *PLoS One* **9**, e87761 (2014).

13. Wang, Y., *et al.* Differential expression of mimecan and thioredoxin domain-containing protein 5 in colorectal adenoma and cancer: a proteomic study. *Experimental biology and medicine* **232**, 1152-1159 (2007).

14. Midwood, K.S., Mao, Y., Hsia, H.C., Valenick, L.V. & Schwarzbauer, J.E. Modulation of cell-fibronectin matrix interactions during tissue repair. *J Investig Dermatol Symp Proc* **11**, 73-78 (2006).

15. Han, S., Khuri, F.R. & Roman, J. Fibronectin stimulates non-small cell lung carcinoma cell growth through activation of Akt/mammalian target of rapamycin/S6 kinase and inactivation of LKB1/AMP-activated protein kinase signal pathways. *Cancer research* **66**, 315-323 (2006).

16. Mikula, M., *et al.* Integrating proteomic and transcriptomic high-throughput surveys for search of new biomarkers of colon tumors. *Functional & integrative genomics* (2010).

17. Spivey, K.A. & Banyard, J. A prognostic gene signature in advanced ovarian cancer reveals a microfibril-associated protein (MAGP2) as a promoter of tumor cell survival and angiogenesis. *Cell adhesion & migration* **4**, 169-171 (2010).

18. Oskarsson, T., *et al.* Breast cancer cells produce tenascin C as a metastatic niche component to colonize the lungs. *Nature medicine* **17**, 867-874 (2011).
